# An efficient drug delivery vehicle for botulism countermeasure

**DOI:** 10.1186/1471-2210-9-12

**Published:** 2009-10-27

**Authors:** Peng Zhang, Radharaman Ray, Bal Ram Singh, Dan Li, Michael Adler, Prabhati Ray

**Affiliations:** 1 Division of Experimental Therapeutic, Walter Reed Army Institute of Research, Silver Spring, Maryland, USA; 2 Cellular and Molecular Biology Branch, Research Division, United States Army Medical Research Institute of Chemical Defense, Aberdeen Proving Ground, Maryland, USA; 3 Department of Chemistry and Biochemistry, University of Massachusetts, Dartmouth, Massachusetts, USA; 4 Neurobehavioral Toxicology Branch, Analytical Toxicology Division, United States Army Medical Research Institute of Chemical Defense, Aberdeen Proving Ground, Maryland, USA

## Abstract

**Background:**

Botulinum neurotoxin (BoNT) is the most potent poison known to mankind. Currently no antidote is available to rescue poisoned synapses. An effective medical countermeasure strategy would require developing a drug that could rescue poisoned neuromuscular synapses and include its efficient delivery specifically to poisoned presynaptic nerve terminals. Here we report a drug delivery strategy that could directly deliver toxin inhibitors into the intoxicated nerve terminal cytosol.

**Results:**

A targeted delivery vehicle was developed for intracellular transport of emerging botulinum neurotoxin antagonists. The drug delivery vehicle consisted of the non-toxic recombinant heavy chain of botulinum neurotoxin-A coupled to a 10-kDa amino dextran via the heterobifunctional linker 3-(2-pyridylthio)-propionyl hydrazide. The heavy chain served to target botulinum neurotoxin-sensitive cells and promote internalization of the complex, while the dextran served as a platform to deliver model therapeutic molecules to the targeted neurons. Our results indicated that the drug delivery vehicle entry into neurons was via BoNT-A receptor mediated endocytosis. Once internalized into neurons, the drug carrier component separated from the drug delivery vehicle in a fashion similar to the separation of the BoNT-A light chain from the holotoxin. This drug delivery vehicle could be used to deliver BoNT-A antidotes into BoNT-A intoxicated cultured mouse spinal cord cells.

**Conclusion:**

An effective BoNT-based drug delivery vehicle can be used to directly deliver toxin inhibitors into intoxicated nerve terminal cytosol. This approach can potentially be utilized for targeted drug delivery to treat other neuronal and neuromuscular disorders. This report also provides new knowledge of endocytosis and exocytosis as well as of BoNT trafficking.

## Background

Botulinum neurotoxins (BoNTs) are produced by the anaerobic *Clostridium botulinum *species of bacteria and are the cause of botulism, a life-threatening neuroparalytic disease. They are extremely potent food poisons, with a mouse LD_50 _of 0.1 ng/kg for type A [1,2]. Aerosol exposure of BoNTs does not occur naturally, but could be attempted by bioterrorists to achieve a widespread effect. It has been estimated that a single gram of crystalline toxin, evenly dispersed and inhaled, could kill more than one million people [2].

BoNTs are large proteins with a molecular weight of 150 kDa. They are produced as a complex containing the neurotoxins and associated proteins [3]. They are synthesized as inactive single chain protoxins and are activated by protease nicking to form a dichain molecule (a 50 kDa light chain (LC) and a 100 kDa heavy chain (HC)) linked through a disulfide bond [4]. The HC is responsible for binding to the target nerve cells (through its C-terminus) and translocating the LC into the cell cytoplasm (through its N-terminus) [5,6].

Inside the neuronal cytosol, the LC acts as a Zn^2+^-endopeptidase against specific intracellular protein targets present either on the plasma membrane or on the synaptic vesicle, and inhibits neurotransmitter release by disabling the exocytotic docking/fusion machinery [5,6]. BoNTs catalyze proteolysis of specific proteins of the soluble NSF attachment protein receptor (SNARE) complex that have been implicated in the exocytotic machinery [5,7]. BoNT/A,/C, and /E cleave a 25 kDa synaptosomal associated protein (SNAP-25).

Current therapy for botulism involves respiratory supportive care and the administration of antitoxin. The antitoxin could be the currently available equine BoNT antibodies or potentially more effective recombinant multivalent antibodies. However, only a few antitoxins, which must be administered before toxins reach the nerve cells, are available. Thus, the therapeutic window for using an antitoxin is short. Once the syndrome is developed, the antitoxin is less effective since it cannot penetrate the nerve cell to neutralize the toxin. The flaccid muscle paralysis caused by BoNT/A lasts for several months [8]. Therefore, patients who have already developed the syndrome must be put under respiratory intensive care during paralysis [1,2,9]. Should a bioterrorist attack occur, public health crisis could arise due to the lack of effective antidotes against botulism, especially in the absence of reliable presymptomatic diagnostics.

For relief from BoNT-mediated paralysis, it is important to rescue the poisoned nerve cells through restoration of the neurotransmitter release process. While drugs have been designed to block the BoNT endopeptidase activity, which is believed to be responsible for the inhibition of neurotransmitter release, delivery of the drugs specifically to the poisoned nerve terminals remains a major hurdle. Therapeutic targeting is important for two main reasons: (a) delivering an effective high concentration of the therapeutic compound to the site of toxicity, i.e., nerve terminals for botulism, and (b) minimizing systemic toxicity, if any, due to treatment compounds. At present, some examples of the proposed pharmacological antidotes for BoNT poisoning are a protease inhibitor, a phospholipase A_2 _activator or a modulator of intracellular free Ca^2+ ^concentration. Since all of these parameters are involved in normal body functions, a systemic therapeutic approach is inadvisable due to potential toxicity concerns.

Therefore, we developed a drug delivery vehicle (DDV) comprising the non-toxic recombinant heavy chain of BoNT-A coupled to a 10-kDa amino dextran via the heterobifunctional linker 3-(2-pyridylthio)-propionyl hydrazide. The heavy chain served to target botulinum neurotoxin-sensitive cells and promote internalization of the complex, while the dextran served as a platform to deliver model therapeutic molecules to the targeted cells.

## Results

### Structure of DDV

**Figure 1:**
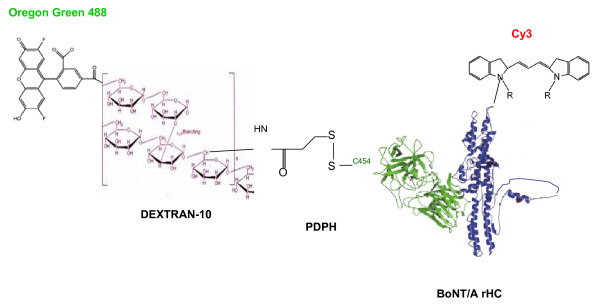
**Schematic representation of the DDV for transport of BoNT/A antagonists**. The schematic representation ofthe DDV construct without drug. The PDPH linker is bound to one of four possible cysteine (C) sulfhydryl groups on the BoNT/A rHC. It is attached to C454, which normally participates in the disulfide linkage with the LC. Cy3 and Oregon green 488 are bound to O-amino groups of lysine in the rHC and dextran, respectively. The dextran is conjugated to the rHC by a C-N bond in one of the glucose residues. In a functional DDV, multiple drug molecules may be attached to dextran carrier.

Initially we designed a DDV utilizing the recombinant BoNT/A heavy chain (rHC), which is known to specifically bind to the presynaptic nerve terminals and be internalized via endocytosis. The DDV construct was a modification of that developed by Goodnough et al. [10] consisting of a targeting molecule, Cy3 labeled purified (from the holotoxin) HC linked by a disulfide bond to a drug simulant, which was Oregon green 488 (OG488) labeled 10 kDa dextran (Fig. 1). The DDV structure was used for further experiments. To our knowledge, this is the first experimental demonstration of a prospective therapeutic approach to treat botulism in a relevant peripheral neuronal model combined with a feasible targeted drug delivery technology.

### rHC was a safe DDV component for delivery of BoNT antidotes

**Figure 2:**
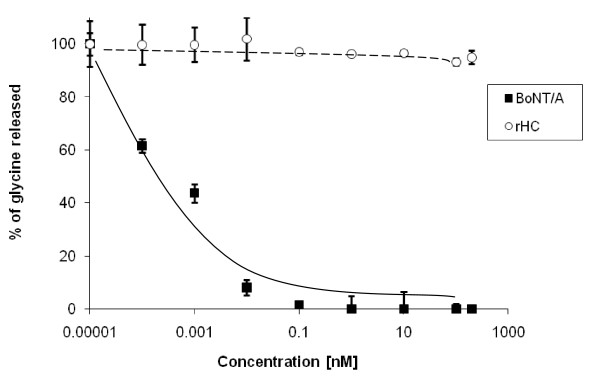
**rHC was a safe DDV component to delivery antidotes**. Primary cultures of mouse spinal cord were exposed to BoNT/A (■) or rHC (○) respectively in indicated concentrations for 16 hrs. Potassium-evoked glycine release was measured. Results expressed as percentage glycine release compared with untreated control. Data points are the mean (± SD) of three separate experiments each determined in triplicate.

To exclude any possible toxicity of the rHC component in our DDV construct, we compared the inhibition of 80 mM K^+ ^stimulated [^3^H]glycine release due to increasing concentrations of rHC or native BoNT/A holotoxin by the assay described in Methods. In these experiments, the results obtained using the particular batch of toxin showed that BoNT/A was quite toxic, as expected, with an IC_50 _(toxin concentration to cause 50% inhibition of neuroexocytosis in untreated control cells) of approximately <1 pM and a total inhibition at ~0.1 nM. However, a much higher concentration of rHC, up to 200 nM, did not show any inhibition of [^3^H]glycine release under the same assay conditions (Fig. 2).

### DDV entry into neurons via BoNT/A receptor mediated endocytosis

**Figure 3:**
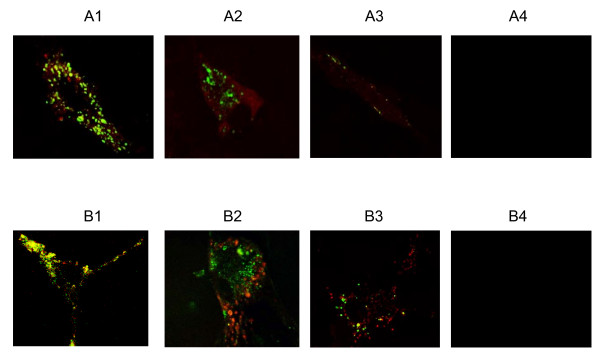
**DDV entry into neurons via BoNT/A receptor mediated endocytosis**. Images in rows A and B were obtained from triplicate cultures exposed for 16 h under the following conditions: (A1) DDV (200 nM) in the absence of rHC; (A2) DDV (200 nM) and rHC (200 nM); (A3) DDV (200 nM) and rHC (600 nM); and (A4) DDV (200 nM) and rHC (2 μM); (B1) DDV (200 nM) in the absence of BoNT/A; (B2) DDV (200 nM) and BoNT/A (200 nM); (B3) DDV (200 nM) and BoNT/A (600 nM); and (B4) DDV (200 nM) and BoNT/A (2 μM). DDV and rHC or BoNT/A were added simultaneously. Note the progressive reductions in fluorescence with increasing concentrations of BoNT/A or rHC. Micrographs were obtained on a Bio-Rad 2000 laser microscope confocal microscope using a 100× oil immersion objective. The fluorescence colors of labelled molecules are: red-rHC; green-OG488-dextran.

The experimental design was to mimic a therapeutic application of the DDV strategy to treat individuals poisoned with BoNT/A and exhibiting clinical symptoms of botulism. Since the targeted DDV approach is based on the premise of a selective entry of DDV into presynaptic nerve terminals via BoNT/A receptor mediated endocytosis, we demonstrated by competition experiments that the uptake of the DDV-Mas-7 was via BoNT/A receptors. In these experiments, 3-week old spinal cord cultures were exposed for 16 hours to DDV (200 nM) alone or to DDV plus a 1-, 3-, or 10-fold excess of unlabeled rHC (Fig. 3, A1-A4) or BoNT/A holotoxin (Fig. 3, B1-B4) added to cultures simultaneously. As seen in Fig. 3, in the absence of rHC or BoNT/A, DDV uptake and dextran separation were as expected; however, a 10-fold excess of rHC or BoNT/A holotoxin completely blocked the uptake of DDV. These results suggested that DDV entry into neurons occurred by the same route as used by BoNT/A.

### Drug carrier could be separated from DDV in a fashion BoNT/A LC dissociates from the HC in the holotoxin

**Figure 4:**
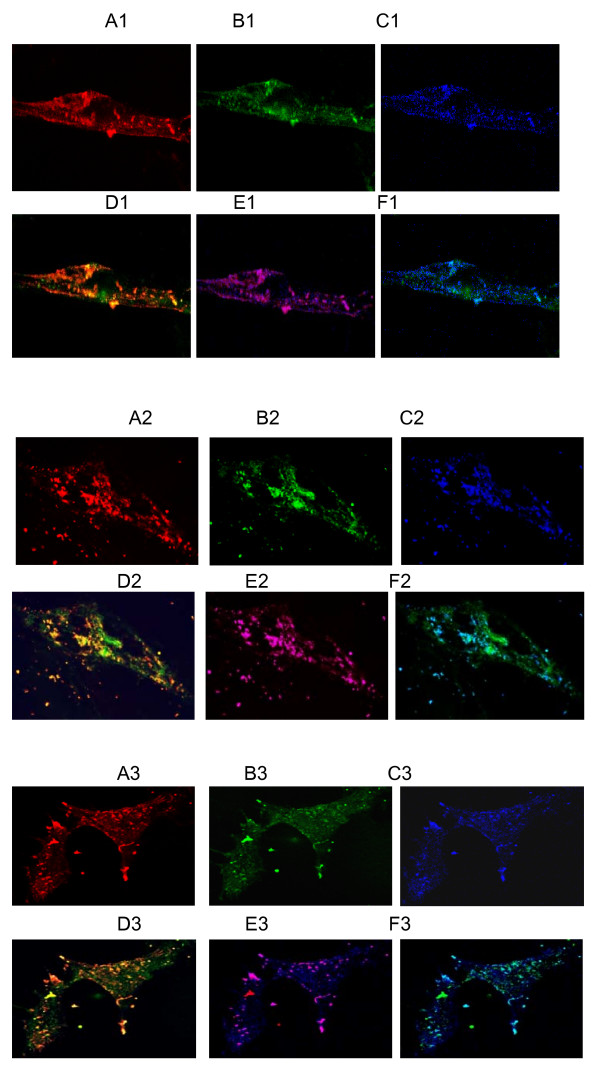
**Separation of the drug carrier molecule in DDV and its translocation into neuronal cytosol is cell maturation dependent**. The figure shows fluorescent images of mouse spinal cord neurons. Cells at different stages of culture were incubated for 16 h with 200 nM solutions of fluorescently labeled DDV, and then labeled with anti-endosome antibody as described under methods. Confocal images shown are as follows: A1-A3, red-rHC; B1-B3, green-OG488-dextran; C1-C3, bright blue-Alexa 633-endosomes; D1-D3, overlay of red and green showing either co-localization (orange) or separation of rHC and dextran; E1-E3, overlay of red and blue showing either the localization (magenta) of rHC in the endosomes or its release into the cytosol, if any; F1-F3, overlay of green and blue showing either localization (light blue or greenish blue) of dextran in the endosomes or its release into the cytosol. The numerical suffixes as in A1, A2 and A3 indicate culture age, i.e., one- (top panels), two- (middle panels) or three-week (bottom panels) old. The results clearly demonstrated that the rHC component of the DDV remained localized in the endosomes, while the OG488-dextran separated from the DDV and migrated into the cytosol.

To determine the efficacy of delivering the therapeutic compound, we studied the separation of the drug carrier from DDV. Confocal microscopy was used to detect the separation of the DDV components. Spinal cord neurons were treated for 16 hours with 200 nM labeled DDV at 37°C. The staining pattern of unseparated DDV was orange (red plus green labeling) and punctate (Fig. 4, D1, D2 and 4, D3). The punctate nature of the staining suggested clustering of DDV in vesicles. The images shown in Fig. 4 highlight the presence of released drug carrier (green) in the particles present in the nerve terminal cytosol. Inclusion of endosome staining (blue) indicated that the DDV was intra-endosomal (Purple, which was red plus blue staining, Fig. 4, E1, E2 and 4, E3) as expected for material transported by BoNT HC. It indicated that the separation of the drug carrier from DDV was in a fashion similar to the dissociation of BoNT/A LC from the HC in the holotoxin.

### Separation of drug carrier from DDV was neuronal maturation-dependent

Different stages of spinal cell culture growth were used to evaluate the efficacy of separation of drug carrier from DDV. Confocal image analysis revealed that about 20, 32 and 40% of the drug carrier component separated from DDV and diffused into the cytosol from endosomes in 1, 2 and 3 weeks culture, respectively (Fig. 4, D1, D2 and 4, D3; table 1). These results indicated that the separation of the drug carrier from DDV is neuronal maturation-dependent. Furthermore, the separations of DDV components occur in a time-dependent manner [see additional file 1, Fig S1].

**Table 1:** The drug carrier separation from DDV is cell maturation-dependent

**Cell growth period**	**1 week**	**2 weeks**	**3 weeks**	
Drug carrier separation rate (%)	20 ± 3	32 ± 5	40 ± 4	

### The processes of exocytosis and endocytosis are not tightly coupled

**Figure 5:**
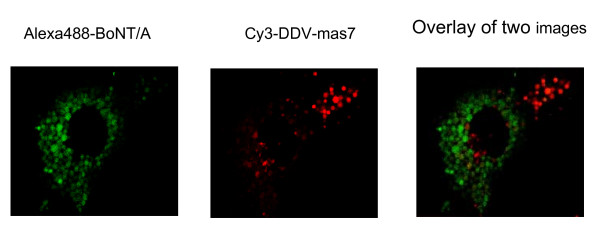
**Exocytosis and endocytosis are not tightly coupled in BoNT/A poisoned neurons**. (a) BoNT/A was labeled with Alexa 488 (green fluorescence). Three week-old cultured mouse spinal cord neurons were incubated with a culture medium containing 1 nM of Alexa 488-BoNT/A for 8 hours at 37°C. Excess Alexa 488-BoNT/A was removed by washings 3 times with fresh culture medium. The cells were incubated with fresh medium for 1 hour and then incubated in medium containing 100 nM Cy3 (red fluorescence) labeled DDV for 16 hours (b). (c) Overlay of red and green showing both BoNT/A (green) and DDV (red) were taken up in the same cell pool, but localized in separate population of endosomes. Fluorescent images were analyzed by Bio-Rad 2000 laser confocol microscope. Alexa 488 was excited at 488 nm line of an argon laser and detected with a 530-nm cutoff filter; Cy3 was excited at 543 nm line of an argon laser and detected with a 565-nm cutoff filter.

It is possible that in neurons, the processes of exocytosis and endocytosis are tightly coupled, i.e., interruption of exocytosis, as in BoNT/A poisoning, might halt endocytosis as well. If true, the DDV approach as presented here would not be a feasible drug delivery system in BoNT poisoned neurons because the uptake of DDV, via endocytosis, could be blocked as a sequel of exocytosis blockade by BoNT. To discount this possibility, we demonstrated uptake of labeled DDV (red fluorescence), of which Oregon green 488 was omitted and and only Cy3 was used, in spinal cord neurons previously exposed to a high concentration (1 nM) of Alexa 488-labeled BoNT-A (green florescence); 1 nM BoNT-A had completely blocked K^+^-stimulated [^3^H]glycine release. To examine DDV-Mas-7 uptake in these cells, the cells were washed once using warm culture medium and reincubated at 37°C with 100 nM DDV for 16 hours. Confocal microscopy results indicated that both BoNT/A and DDV were taken up in the same cell pool, but localized in separate population of endosomes (Fig. 5), demonstrating internalization of DDV via endocytosis into BoNT/A poisoned neurons. This suggested that the exocytosis and endocytosis are not tightly coupled in BoNT/A poisoned neurons.

## Discussion

Primary cultures of spinal cord represent a convenient and sensitive system to study mechanisms of neurotransmitters release [11,12]. Internalized neurotransmitters by spinal cord neurons in culture were released quantitatively in response to depolarization and Ca^2+^. This release is inhibited by tetanus toxin and botulinum neurotoxins in a concentration- and time-dependent manner [13-17]. Therefore, this system serves as a suitable model to examine the efficacy of prospective BoNT countermeasures. Sheridan and Adler indicated that the evoked release of neurotransmitters, notably glycine, in this system was time-dependently increased [18]. In our studies, there was a pronounced time-dependent increase of the drug carrier separation from DDV, which paralleled an enhancement of transmitter release.

We postulate that the ability of drug carrier to separate from DDV in our spinal cord cell culture model is dependent upon neuronal maturation at increasing age of the cultures. Regarding DDV uptake and processing in neurons, the results in Fig 4 clearly demonstrated the following facts. The red (Cy3-rHC) (A1-A3) and the blue (Alexa 633-endosome) (C1-C3) fluorescence signals were strongly present in neurons at any stage of development, however, were always in a distinct punctate localization. Moreover, the red and the blue signals were always co-localized (E1-E3) and the red signals never diffused into the cytosol. These observations indicated that the DDV was readily internalized into neurons apparently via endocytosis and the rHC component remained in the endosomes as generally believed to be the case in BoNT endocytosis and trafficking phenomena. The HC remains localized in the endosome and not released into the cytosol. These micrographs exhibit both punctuate and diffused distribution of signals with the level of diffusion apparently increasing with increased age of cultures. This observation may be explained as follows: in mature neurons, the DDV is taken up into endosomes, the dextran component separates from the rHC and then gets released into the cytosol. This explanation is supported by our results presented in the micrographs showing the overlays of red and green fluorescence (D1-D3) and those of green and blue fluorescence (F1-F3). In D1-D3, overlap of red and green generated orange indicating intact DDV; the released OG488-dextran was green. It should be noted that the orange was punctate suggesting endosomal localization, whereas the green was diffuse suggesting the release of OG488-dextran component of the DDV into the cytosol, which was enhanced with increasing age of cultures. Using the images in D1-D3, we calculated the separation and release rates of OG488-dextran from the DDV by utilizing the Bio-Rad AutoDeblur and AutoVisualize software to quantitate fluorescence intensity. The separation/release rates were expressed as 100% (total in images) minus percentage of co-localization rate (Table 1). In F1-F3, overlap of bright blue and green generated light or greenish blue indicating OG488-dextran remaining in the endosomes; the released OG-dextran was green. On examining the D1-D3, E1-E3 and F1-F3 micrographs, it is interesting to note that intact DDV (D1-D3), rHC (E1-E3) and unreleased dextran all seem to be localized in the same endosomal pool; this provides additional support to our proposed mechanism of DDV uptake and processing. Relevant to this proposition, most significant was our demonstration that in neurons, the DDV function that required an efficient endocytotic mechanism was developmentally regulated, i.e., neuronal maturation-dependent. Moreover, our results showed that the efficiency in neuroexocytosis was also a function of mature neurons.

To demonstrate the feasibility of delivering a therapeutic compound via the DDV, we examined the separation of the prototype drug carrier dextran from DDV. The results indicated that the drug carrier components were satisfactorily separated from DDV and diffused into cytosol. As described in the results section, the targeting component of the DDV, i.e., rHC was nontoxic. Therefore, the DDV approach presented here may be a physiologically compatible and a feasible targeted drug delivery method to counteract botulism.

Although it was believed to be the case, our results provided the first experimental evidence that the HC of BoNT/A upon internalization into neurons remains localized in the endosomes and thus, does not participate in the cytosolic mechanism of BoNT/A toxicity. Very meaningful was the fact that BoNT/A poisoned neurons, which were totally incapable of stimulated exocytosis, could still incorporate the HC via endocytosis. This was apparently due to new or spare receptors available for HC molecule binding on the plasma membrane. Furthermore, the exocytosis and endocytosis phenomena in neurons may not necessarily be coupled tightly. Neale et al. also reported that BoNT/A blocked synaptic vesicle exocytosis but not endocytosis at nerve terminal [20].

In conclusion, this report provides new knowledge of endocytosis and exocytosis, as well as of BoNT trafficking and action. Notably, application of this DDV approach to antagonize botulism is not necessarily limited to the neuronal targeting of BoNT as shown here, but also should be useful for delivery of other prospective antidotes, such as protease inhibitors to protect the vesicle fusion proteins as applicable. Finally, the success in the DDV strategy against botulism shown here may open new avenues in developing technologies to treat other neurological disorders that require a targeted delivery of therapeutics to affected neurons or tissues. Before the actual studies are conducted, we can only speculate on a possible route of administration of the DDV as a therapeutic in a patient. Oral or inhalation routes are inadvisable. The oral administration may result in DDV degradation in the gastrointestinal system. The inhalation administration may result in a slower DDV absorption and may also require a high DDV concentration, which could possibly trigger a cell-mediated immune response. Based on these considerations, we propose the intravenous route which should rapidly achieve a high DDV level in the circulation for an effective drug delivery into BoNT poisoned nerve terminals.

## Conclusion

An effective botulinum neurotoxin-based drug delivery vehicle can be used to directly deliver toxin inhibitors into the intoxicated nerve terminal cytosol. The concept may possibly be utilized for drug delivery for other neuronal and neuromuscular disorders. Besides a BoNT therapeutic approach, this report also provides new fundamental knowledge of endocytosis and exocytosis as well as of BoNT trafficking in neurons.

## Methods

### Construct a model drug conjugated drug delivery vehicle

We designed a DDV utilizing recombinant BoNT/A heavy chain (rHC), which is known to specifically bind to the presynaptic nerve terminals and be internalized via endocytosis. The DDV construct was a modification of that developed by Goodnough et al., [10]. The DDV consisted of a targeting molecule, Cy3 labeled rHC linked by a disulfide bond at Cys454 of rHC to a drug simulant, Oregon green 488 (OG488) labeled 10 kDa dextran (Fig. 1). The DDV construct was soluble in aqueous medium and was stable under our experimental conditions, i.e., at 37°C.

### Spinal cord cultures

Timed pregnant C57BL/6NCR mice were obtained from the Frederick Cancer Research and Development Center (Frederick, MD). Research was conducted in compliance with the Animal Welfare Act and other federal statutes and regulations relating to animals and experiments involving animals and adheres to principles stated in the *Guide for the Care and Use of Laboratory Animals*, NRC Publication, 1996 edition. Spinal cords were removed from fetal mice at gestation day 13. Cells were dissociated with trypsin and plated in collagen-coated 4 well coverslips or 35 mm diameter 6-well culture plates at a density of 10^5 ^cells/cm^2^. Cells were grown in Eagle's Minimum Essential Medium with 5% heat-inactivated horse serum and a nutrient supplement (N3) at 37°C in 90% air/10% CO_2_. Cell cultures were treated with 54 mM 5-fluoro-2-deoxyuridine and 140 mM uridine from day 5-9 after plating to inhibit glial proliferation. Cultures were fed 1-2 times per week and were used for experiments at 1 to 3 weeks after plating.

### ^3^[H]glycine release assay

^3 ^[H]glycine release was determined by a modification of the method described by Williamson et al. [19]. Spinal cord cells were incubated at 37°C for 30 min in HEPES-buffered saline (HBS) containing 2 mCi/ml ^3 ^[H]glycine. The cells were washed briefly with Ca^2+^-free HBS and incubated sequentially for 7 min in each of the following modified HBS solutions: 5 mM KCl/0 mM Ca^2+^, 80 mM KCl/2 mM Ca^2+ ^and 5 mM KCl/0 mM Ca^2+^. Each incubation solution was collected, and the radioactivity was determined by scintillation counting.

### Uptake of DDV by spinal cord neurons and release of dextran

Cells were exposed to DDV, Cy3-labeled rHC, or Oregon green 488-labeled dextran in growth medium for 16 h at a concentration of 200 nM at 37°C. Cells were subsequently washed three times with growth medium and fixed overnight using 2% paraformaldehyde. The coverslips containing fixed cells were mounted between a glass slide and glass coverslip and viewed on a Bio-Rad 2000 laser confocal microscope. Oregon green 488 was excited at 488 nm and read through a 515-nm cutoff filter. Cy3 was excited at 543 nm and read through a 565-nm cutoff filter. To minimize photobleaching, Slowfade Light was added to the mounting medium. Micrographs were obtained using a Bio-Rad laser confocal microscope with a 100× oil immersion objective. Images were collected with Bio-Rad software. Co-localization of rHC and dextran and separation rates of dextran from DDV were then calculated by utilizing the Bio-Rad AutoDeblur and AutoVisualize software to quantitate fluorescence intensity. Separation rates were expressed as % total minus % co-localization.

### Determination of rHC localization

Experimental procedures were similar to those described above under "Uptake of DDV by spinal cord neurons and release of dextran", except that after fixing with 2% paraformaldehyde, cells were subsequently washed three times with D-PBS and permeabilized with 0.2% TritonX-100 for 10 min in room temperature (RT). Cells were blocked with 4% BSA in D-PBS at 4°C for 1 h and subsequently incubated with goat anti-EEA1 antibody at RT for 1 hr. After wash five times with D-PBS, cells were incubated with a secondary antibody (donkey anti goat-Alexa 633) at RT for 0.5 h. Cells were subsequently washed three times with D-PBS. The coverslips containing fixed cells were mounted between a glass slide and glass coverslip and viewed on a Bio-Rad 2000 laser confocal microscope. Excition and emmission for Cy3 and OG-488 were as stated above. Alexa 633 was excited at 632 nm and detected with a 649 nm cutoff filter. Micrographs were obtained using a Bio-Rad laser confocal microscope with a 100× oil immersion objective.

## List of Abbreviations

BoNT: Botulinum neurotoxin; LC: light chain; HC: heavy chain; DDV: drug delivery vehicle.

## Competing interests

The authors declare no competing financial interests. The opinions or assertions contained herein are the private views of the author, and are not to be construed as official, or as reflecting true views of the Department of the Army or the Department of Defense.

## Authors' contributions

PR and RR conceived the project, guided research directions, and edited the manuscript. RR provided special advice on neurobiological issues. PR procured the funding and served as the principal investigator being responsible for overall supervision and all reporting requirements. PZ performed the experiments, analyzed data, drafted and revised the manuscript. BRS supervised all synthetic chemistry work and provided the products to include all unlabeled and labelled botulinum toxin heavy chain, drug simulant and the delivery vehicle molecules. BRS also served as an expert consultant for the project and provided advice on experiments. DL performed some initial experiments and analyzed data. MA served as an expert consultant for the project and edited the manuscript. All authors read and approved the final manuscript.

## Supplementary Material

Additional file 1 **Fig. S1. Fluorescent images of mouse spinal cord neurons demonstrating that the separations of DDV components occur in a time-dependent manner**. Three weeks old cultured cells were incubated for 1 h (A), 12 h (B) and 24 h (C) with 200 nM solutions of fluorescently labeled DDV. Confocal images shown are as follows: A, red-rHC that fluorescence elicited at an excitation wavelength of 543 nm; B, green-OG488-dextran that fluorescence elicited at an excitation wavelength of 488 nm. The micrographs represent overlays of the two images, red and green. Separation of dextran from rHC is observed at 12 h or earlier. Click here for file
