# Supplementary figures and images for: An efficient drug delivery vehicle for botulism countermeasure
Source: BMC Pharmacol. 2009 Oct 27;9:12. doi: 10.1186/1471-2210-9-12 (PMC2774289; doi:10.1186/1471-2210-9-12)

## Slide 1
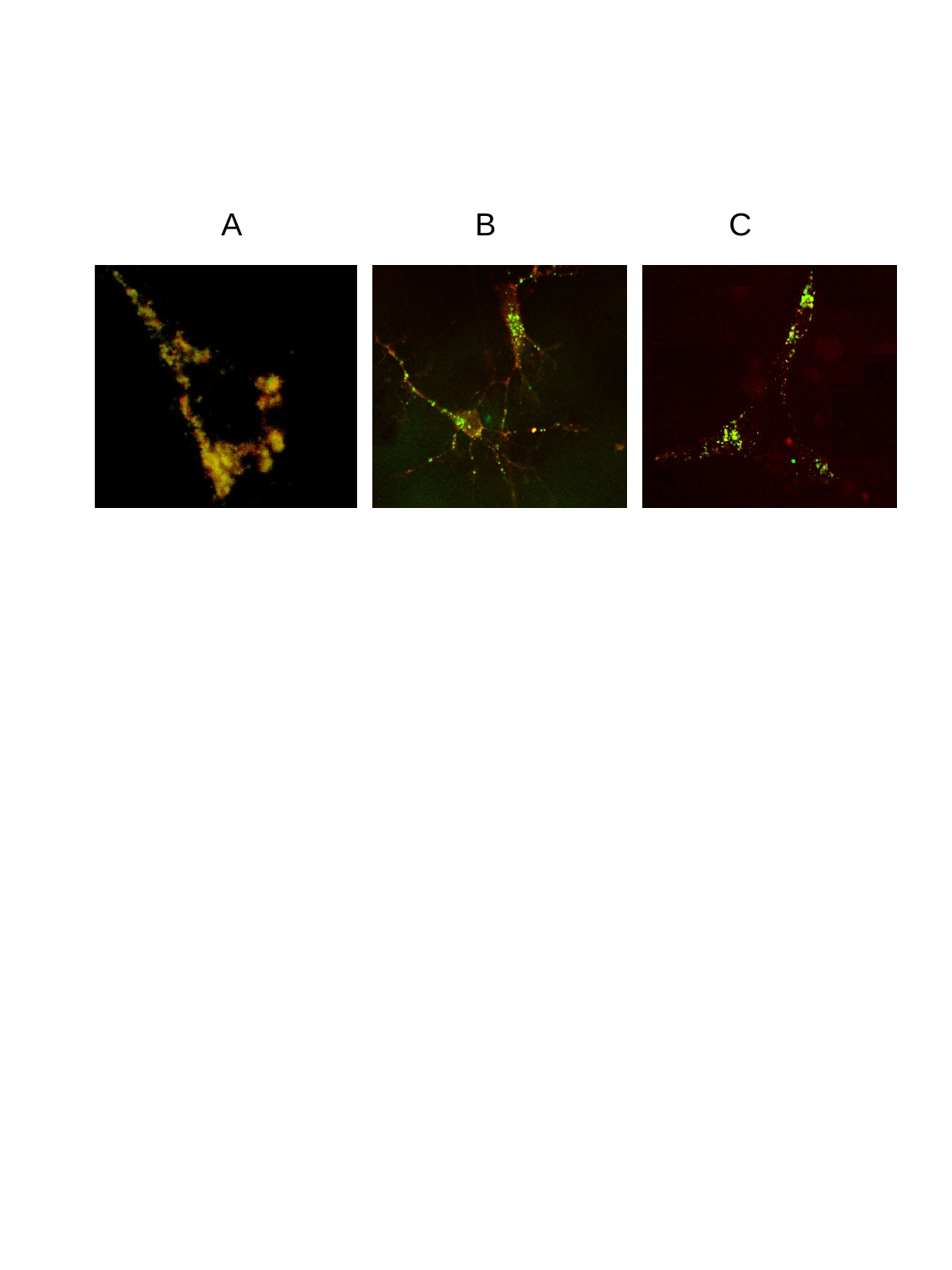

A		B		C

Supplement: Additional file 1 — Fig. S1. Fluorescent images of mouse spinal cord neurons demonstrating that the separations of DDV components occur in a time-dependent manner. Three weeks old cultured cells were incubated for 1 h (A), 12 h (B) and 24 h (C) with 200 nM solutions of fluorescently labeled DDV. Confocal images shown are as follows: A, red-rHC that fluorescence elicited at an excitation wavelength of 543 nm; B, green-OG488-dextran that fluorescence elicited at an excitation wavelength of 488 nm. The micrographs represent overlays of the two images, red and green. Separation of dextran from rHC is observed at 12 h or earlier. [file 1471-2210-9-12-S1.PPT]
